# Satisfaction with web-based training in an integrated healthcare delivery network: do age, education, computer skills and attitudes matter?

**DOI:** 10.1186/1472-6920-8-48

**Published:** 2008-10-15

**Authors:** Ashish Atreja, Neil B Mehta, Anil K Jain, CM Harris, Hemant Ishwaran, Michel Avital, Andrew J Fishleder

**Affiliations:** 1 Cleveland Clinic Foundation, Cleveland, Ohio 44195, USA; 2 University of Amsterdam, 1018WB Amsterdam, the Netherlands; 3 Medical Informatics Fellowship, Department of General Internal Medicine, Desk A-91, Cleveland Clinic, 9500 Euclid Ave., Cleveland, OH 44195, USA

## Abstract

**Background:**

Healthcare institutions spend enormous time and effort to train their workforce. Web-based training can potentially streamline this process. However the deployment of web-based training in a large-scale setting with a diverse healthcare workforce has not been evaluated. The aim of this study was to evaluate the satisfaction of healthcare professionals with web-based training and to determine the predictors of such satisfaction including age, education status and computer proficiency.

**Methods:**

Observational, cross-sectional survey of healthcare professionals from six hospital systems in an integrated delivery network. We measured overall satisfaction to web-based training and response to survey items measuring Website Usability, Course Usefulness, Instructional Design Effectiveness, Computer Proficiency and Self-learning Attitude.

**Results:**

A total of 17,891 healthcare professionals completed the web-based training on HIPAA Privacy Rule; and of these, 13,537 completed the survey (response rate 75.6%). Overall course satisfaction was good (median, 4; scale, 1 to 5) with more than 75% of the respondents satisfied with the training (rating 4 or 5) and 65% preferring web-based training over traditional instructor-led training (rating 4 or 5). Multivariable ordinal regression revealed 3 key predictors of satisfaction with web-based training: Instructional Design Effectiveness, Website Usability and Course Usefulness. Demographic predictors such as gender, age and education did not have an effect on satisfaction.

**Conclusion:**

The study shows that web-based training when tailored to learners' background, is perceived as a satisfactory mode of learning by an interdisciplinary group of healthcare professionals, irrespective of age, education level or prior computer experience. Future studies should aim to measure the long-term outcomes of web-based training.

## Background

Large healthcare facilities are required to educate their workforce about various regulations and to document this training. Initiatives like the Privacy Rule of the Health Information Portability and Accountability Act (HIPAA) and the National Patient Safety Goals of the Joint Commission on Accreditation of Healthcare Organizations (JCAHO) are just two recent examples. In addition, health care professionals require training on equipment, skills and software. Using traditional methods such as instructor-led classes to train a large and diverse workforce is time consuming, expensive and labor-intensive. Web-based training can potentially overcome these limitations [1-5].

Web-based training (also variably referred to as 'online training' or 'computer-based learning' or 'e-learning') provides learners with 24-hour access to the training courses, is self-paced, eliminates the need to travel, is less disruptive for the work schedule, and can decrease the time associated with learning by as much as 25 to 30% [2-4]. Moreover, it can substantially save time for the faculty and instructors since web-based training can be developed once and delivered multiple times across various locations. Published studies evaluating web-based education and training have shown that web-based education is at least as effective as traditional education, that it is likely to be more efficient and that learners enjoy it more [6-12]. These advantages make web-based training a very attractive option for training healthcare personnel efficiently and effectively [13]. Furthermore, many hospitals and clinics are upgrading their information technology infrastructure as they increasingly adopt electronic health records. This infrastructure can also support the deployment of web-based training.

However, most of the literature supporting web-based education and training involves small groups of learners with similar backgrounds and good computer skills, such as students attending off-campus programs or professionals attending web-based continuing medical education (CME). Published literature has also been narrowly focused on comparing achievement scores with limited attention paid to learner adoption and satisfaction [10,14-18]. Thus, it is difficult to generalize these findings to the training of a diverse healthcare workforce [14,15]. Is web-based training suitable for all learners with different learning styles, education levels, computer skills and attitudes towards technology [14,19]? This question must be addressed before web-based training is adopted on a large scale.

HIPAA Privacy Rule requirements provided us with a unique opportunity to implement and evaluate web-based training on a large scale. It required compliance to federal privacy standards from all healthcare organizations that maintain or transmit electronic healthcare-related information, including physician offices, hospitals, health plans, and healthcare clearinghouses [20-23]. In order to streamline training across our integrated delivery network (IDN), which is composed of a large tertiary-care academic center and its affiliated hospital systems, we developed a web-based HIPAA course for healthcare professionals including physicians, clinical researchers, pharmacists, nurses, secretarial staff, nutritionists and trainees in these fields. The project was developed in collaboration between the Center for Online Medical Education and Training and the Information Technology Division of the Academic Center.

## Methods

### Content development

The course content and the delivery platform for web-based training were developed in parallel. An institutional committee identified the course objectives and 15 key policies for training employees on the HIPAA Privacy Rule. These served as the blueprint for an instructional design team that created lessons based upon the storyboard plan. The lessons were then developed into online media and assigned to employees with different training roles. The first role was developed for clinicians such as nurses, staff physicians, pharmacists, nutritionists and house staff. The second was for administrators and the third was for all other employees exposed to protected health information (PHI). We used Macromedia Authorware^® ^6.0 (Macromedia, Inc., San Francisco, CA) software to create the interactive content. The final course grouped the 15 policies into 5 lessons: PHI, privacy practices, patient rights, specialty groups and HIPAA in research.

The lessons were designed in a series of scenarios using several familiar interactive modalities including "drag and drop," "multiple choice questions" and "hotspots". Specific details on these interactive modalities have been published by authors elsewhere [24]. Each lesson's specific content was dynamically altered to reflect the user's role. Case scenarios were customized for specific job roles, i.e., users with a "clinical" role would see a scenario different from those with an "administrator" role. This ensured that the training was relevant to the employee's job function. The entire course was designed to take approximately 45 minutes to complete.

### Course delivery

We designed and deployed a web-based learning portal on our institutional intranet to deliver the training course. To track compliance with the mandatory training, all users were authenticated through their employee number and secure password against our Human Resources (HR) employee database. The database also helped us assign users to appropriate training roles (i.e. clinician, administrator, or other) based on their job profiles.

Once the entire course was designed and tested, we informed the employees through our institutional intranet, employee newsletters and administrative supervisors for each department. The course was accessible from any of the networked computers at the various facilities including hospitals, ambulatory clinics, library and administrative areas. We tracked course completion statistics for individuals and departments and sent periodic reminders to employees and supervisors to meet the deadline set forth in the HIPAA Privacy Rule.

### Instrument design

At the completion of the course, all users were asked to complete a confidential voluntary online survey. The Cleveland Clinic Institutional Review Board (IRB) waived the requirement for informed consent as the survey involved "no more than minimal risk" to the respondents. The survey was designed to determine satisfaction with web-based training for employees from different organizations in our IDN and to assess potential predictors of satisfaction with web-based training. We designed our survey based on modification of a previously validated survey instrument on web-based education [17]. Most questions were scored on a 5-point Likert scale (1 = strongly disagree, 2 = disagree, 3 = neither agree not disagree, 4 = agree, 5 = strongly agree). Information on user demographic characteristics (age, gender, and race) was retrieved from the HR employee database in a de-identified manner. The first 700 survey respondents were used to pilot our instrument.

### Statistical analysis

We used a two-step approach comprised of exploratory and confirmatory factor analyses to identify the underlying constructs of the survey, as previously reported by the authors [25]. The final instrument was determined to be reliable with high alpha coefficients for each of the five distinct constructs in addition to Course Satisfaction: Website Usability (3 items; α = 0. 806), Course Usefulness (2 items; α = 0.808), Instructional Design Effectiveness (5 items; α = 0.922), Computer Proficiency (3 items; α = 0.9015) and Self-learning Attitude (3 items; α = 0.849). Website Usability comprised items on the appeal of web site design, ease of navigation and the ability of web pages to load quickly. Course Usefulness elicited information on whether the course was relevant and helped improve understanding of the subject. Instructional Design Effectiveness asked for the respondents' opinion about the effectiveness of instruction methodologies and interactivity in learning the course content. Self learning Attitude items asked the respondents to indicate their motivation to learn new topics, preference for active learning and ability to learn on their own. Computer Proficiency items asked learners to rank their computer skills, experience and comfort level in using computers. Psychometric analysis of the survey instrument is reported by the authors elsewhere [25].

The constructs identified by factor analysis along with the demographic variables were tested univariately to assess significance with overall satisfaction using the Kruskal-Wallis test [26]. The Kruskal-Wallis test is a method of testing the hypothesis that several populations have the same continuous distribution of an underlying variable. To apply this method to the continuous predictor "age," the variable was converted to five categories based on percentiles. In all cases, distribution-free p-values were computed via permutations [27]. All variables were found to be significant (p < 0.001) except for gender. All predictors were then tested in a multivariable ordinal regression model with overall satisfaction used as the ordinal response Y variable. The model used was a proportional odds ordinal logistic regression model and was fit using maximum likelihood estimation. Implementation was via the lrm() function of the Design Library in R [28].

To further validate the findings, a bootstrap reproducibility analysis was performed as follows. For each of 10,000 bootstrap samples, a multivariable ordinal regression model was computed. The average number of times a variable was found to be significant at a 0.01 level was determined from the 10,000 model fits. A variable was defined as bootstrap reproducible if this average was > 0.99. Bootstrap reproducibility is a useful measure in large data sets like ours. Due to the large sample sizes involved, hypotheses can be rejected on the basis of very small differences in test statistics, yet often these differences are of little relevance in a scientific context. Of the variables found significant in the original analysis, 4 item sets were deemed to be bootstrap significant. No demographic variable was found to be bootstrap significant. To see the specific influence of individual survey items within a construct, a Random Forests Classification and Regression Trees (CART) method was fit using the original survey questions from our instrument [29]. The procedure was implemented using the random Forest Library in R, with all default choices selected. An out-of-bagged estimate of prediction showed that Random Forests achieved a 29% misclassification rate.

## Results

### Respondent characteristics

**Figure 1:**
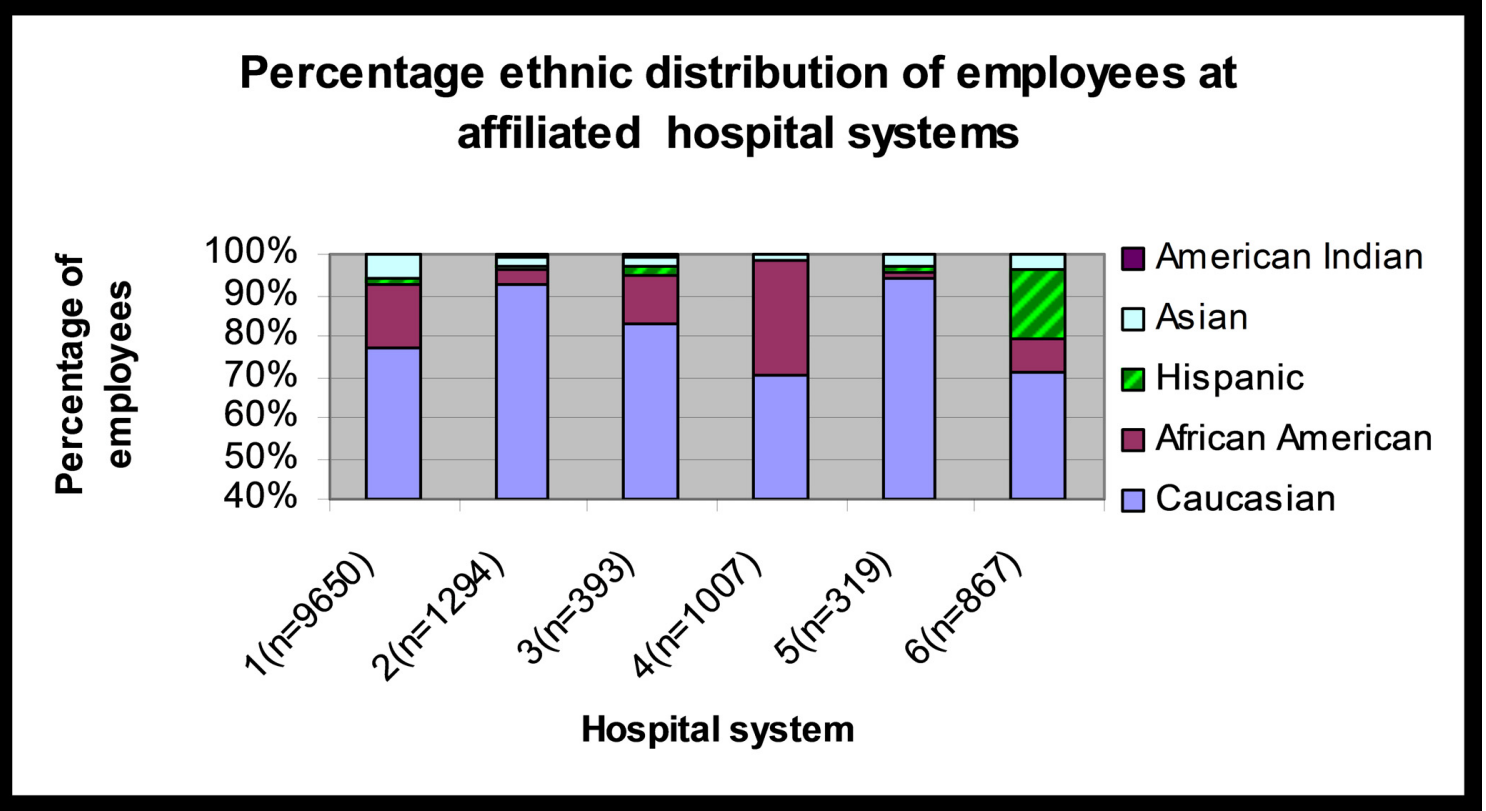
Ethnic distribution of employees at the affiliated hospital systems (n = 13,530).

A total of 17,891 employees took the web-based course over a two month period. Of these, 13,537 completed the survey, for an overall response rate of 75.6%. When compared to respondents, non-respondents were more likely to be female (80.1% vs. 76.1%), older (42.9 years vs. 40.5 years) and non-Caucasian (29.0% vs. 21.6%). Table 1 summarizes the demographic characteristics of the respondents. Most of the respondents (92.3%) had used a computer before but only 50% had,, previously participated in web-based training. There was a good representation of employees with secondary education or more (high school, bachelors, masters, doctorate and others). Figure 1 shows the ethnic distribution of the respondents across the six hospital systems. Hospital 1 and hospital 4 had a higher percentage of African-American employees whereas hospital 6 (located in Florida) had more Hispanic employees.

**Table 1:** Demographics of the respondents

**Characteristics**	**Employees **(n = 13,537)
Mean age, years (range)	40.5 (15 to 78)
Female gender, %	76.1
Race, %	
African American	14.4
American Indian	0.2
Asian	4.6
Caucasian	78.4
Hispanic	2.4
Not Reported	0.1
Education level N = 11 898, %	
No high school diploma	0.1
High school diploma	22.3
Bachelor's degree	33.7
Master's degree	8.6
Doctorate degree	15.3
Other	20.0
Computer usage, %	
Daily	82.4
More than once a week	7.9
Weekly	3.5
Occasionally	5.5
Never	0.7
Past participation in online training, %	
Yes	50.0
No	44.0
Don't know/not sure	6.0
Site of accessing online course, %	
Home	9.7
Office/Clinical workstation	80.2
Library	3.7
Other	6.3

### Satisfaction with web-based training

**Figure 2:**
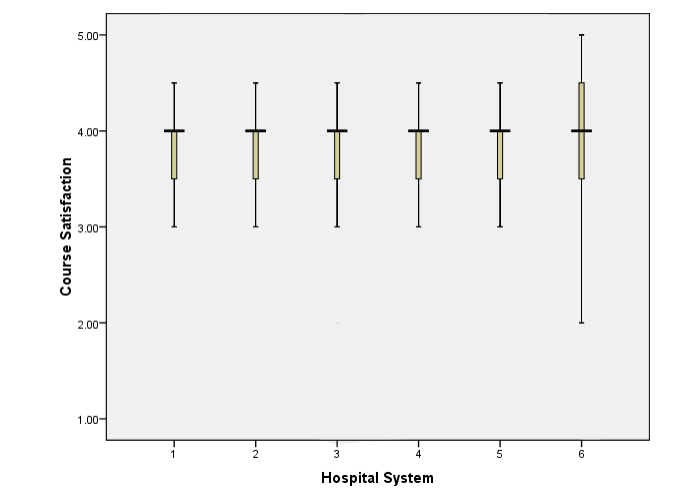
Graph illustrating the median score and interquartile range of satisfaction with web-based training across the six hospital systems of the integrated delivery network.

Overall course satisfaction was measured by the survey item "I am satisfied with the online course." The median value for overall satisfaction was 4 on a scale of 1–5 (interquartile range, 3.5 to 4.0). Overall, 76.1% of the respondents rated themselves as satisfied or very satisfied (rating 4 or 5) and 64.6% preferred web-based training over traditional instructor-led training (rating 4 or 5) 19.5% were neither satisfied nor dissatisfied with online course (rating 3) whereas 4.4% expressed dissatisfaction (rating 1 or 2). Univariate analysis did not show any significant difference in satisfaction with gender or education. The results were similar when data from each different health system was analyzed separately (Figure 2).

### Multivariable analyses

At the 0.01 level of significance and using a 0.99 bootstrap reproducibility measure, multivariable ordinal regression found that all the identified constructs except Computer Proficiency were significant predictors (Table 2). Importantly, we found no non-instrument specific predictor to be significant. In particular, neither gender, age, education nor race was found to be predictive.

**Table 2:** Summary of ordinal regression analysis with satisfaction as the dependent variable

**Item set**	**Regression Coefficient**	**Standard Error**	**Wald Z**	***P *value**	**Boot Percent**
Instructional Design	1.186	0.036	33.409	<0.001	1.000
Web Usability	0.479	0.034	14.258	<0.001	1.000
Course Usefulness	0.662	0.034	19.292	<0.001	1.000
Learning Attitude	0.367	0.038	9.613	<0.001	1.000
Computer Proficiency*	0.133	0.034	3.965	<0.001	0.918

Adjusted R^2 ^= 0. 401, n = 13,537, *p *< 0.0001

* Computer Proficiency had less than 0.99 bootstrap reproducibility. Age, gender, education level, race and hospital system were not found to be significant predictors

**Figure 3:**
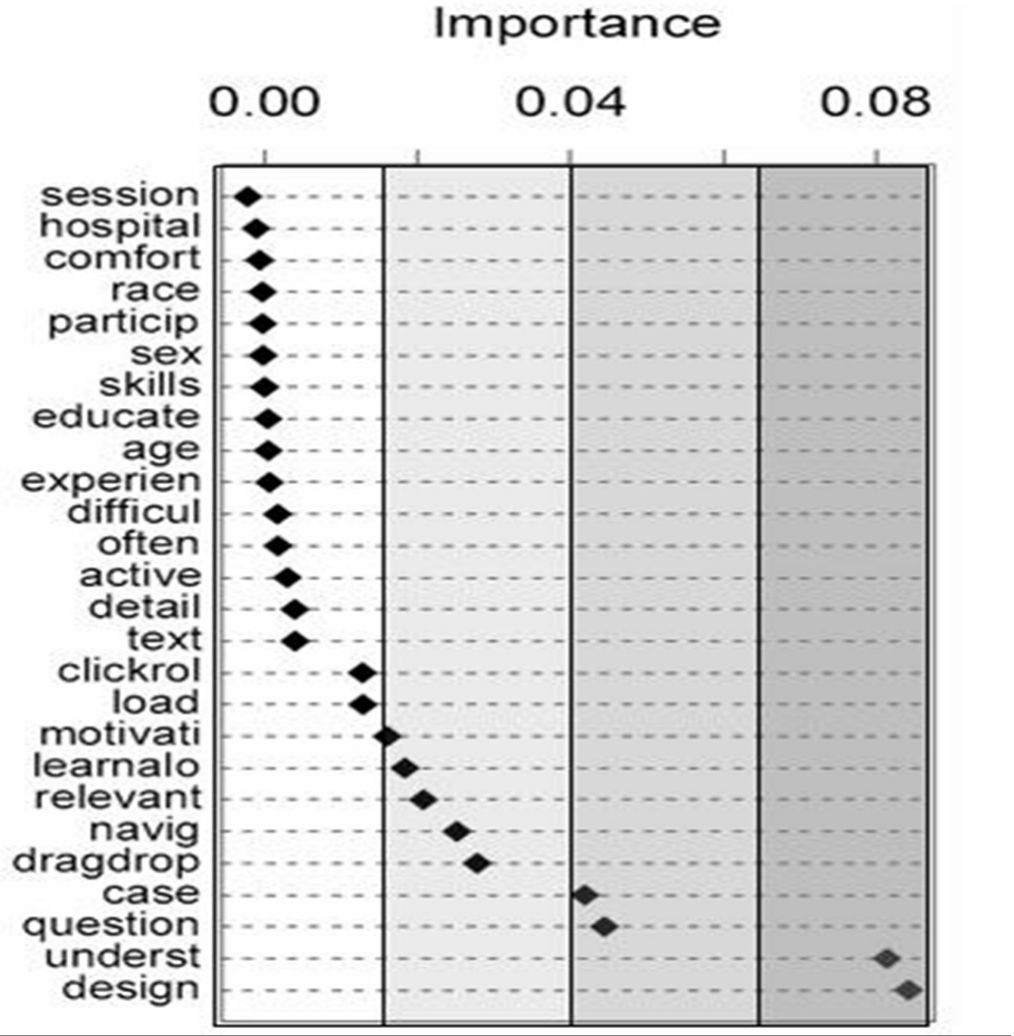
**The influence of survey items in predicting satisfaction**. The influence of individual survey items in predicting satisfaction to web-based training as found by random forests classification and regression trees (CART) method.

Random Forests helped identify the separate effects of the survey questions within the facet of the five constructs. As seen in Figure 3, we can rank the influence of individual survey items into one of the four categories: highly influential, very influential, moderately influential and less influential. Included in the highly influential category were the items: Appeal of the web site design (item set – Web Usability) and the ability of the course to improve understanding of the subject (item set – Course Usefulness). In the very influential category were the Instructional Design Effectiveness items that rated the effectiveness of question/answers and case-based scenarios (question, case). In the moderately influential category were other Instructional Design Effectiveness items (drag-drop, click-roll); Web Usability items (navig, load); Course Usefulness item (relevant); and Self-learning Attitude items (learnalone, motivation). Demographic items (age, gender, education or race) and Computer Proficiency items were less influential.

## Discussion

Training healthcare professionals is not an easy task due to inherent characteristics such as shift work, moderate to high employee turnover, and the difficulty in organizing group-training sessions due to conflicts with clinical responsibilities [13,30]. This large-scale study demonstrates that web-based training can be deployed for healthcare workforce spread over different geographic areas, without compromising learner satisfaction. We were able to train about 18,000 professionals, including physicians, researchers, pharmacists, nurses, secretarial staff, nutritionists and trainees in these fields, across different hospital systems within a short span of two months. Over three-quarters of the respondents were satisfied or very satisfied with web-based training and most of them felt that the course was relevant and helpful for enhancing their understanding of the subject.

Multivariable analyses revealed that the constructs Instructional Design, Web Usability and Course Usefulness item sets were better predictors of satisfaction with web-based training than Computer Proficiency or demographics. Random Forests revealed that the two survey items: "Appeal of the web site design" and the "Ability of the course to improve understanding of the subject" were highly influential. Demographic factors were not significant. We tried to increase understanding of the subject by using sound instructional methods (such as real-life case scenarios) that matched learners' needs and educational background. Our results are consistent with past literature suggesting that perceived quality of instruction most reliably predicts satisfaction in a technology-mediated course and that poorly designed educational programs or materials are not improved by being presented on a web page [15,31]. Technology can *facilitate *but does not *replac*e sound instructional methods [18].

One of the main strengths of our study is that we evaluated a broad-spectrum of employees with varying levels of education and computer skills. There are many successful studies on web-based training reported in the literature but most of these evaluated students in a special environment such as university campuses or were restricted to computer-savvy professionals in certain specialties or settings (such as web-based CME) [7,8,32,33]. This limits their adoption and generalizability to other settings and has led to the prevailing notion that web-based learning is an effective tool only for people with advanced education and adequate computer skills [14,15,18,19]. Our study disproves this notion and shows that health care professionals at all levels of education can be equally satisfied with web-based training if it is designed and tailored to their job profile. We had a limited number of respondents with less than high-school education (n = 14) and this precludes us from drawing any conclusions for this group. However, a smaller study on web-based training among healthcare employees representing departments such as housekeeping, food and nutrition, and facilities maintenance found that even computer-naïve employees can successfully negotiate web-based training [30].

In addition to time savings and enhanced 24-hour access to courses, web-based training can also yield a good return on investment (ROI). We utilized existing internal resources to train nearly 18,000 employees spread across different hospital systems which helped us keep our development and delivery costs to a minimum. Although other institutions may require additional resources and expense, web-based training can still prove to be cost-effective when compared to traditional learning methods [34]. Blair describes how an IDN used the web-based lessons developed by a commercial vendor to train their 7800 employees [30]. The web-based training substituted for 500 individual classroom sessions and helped save around $400,000 and 14,000 employee hours. Unfortunately, the report did not include any formal evaluation of the training and hence prevents us from drawing any conclusions regarding satisfaction. Other studies suggest that web-based training can reduce up to 70% of employers' training budgets by eliminating employee travel from off-site locations, cost of updating printed materials, and reducing the amount of time that employees spend overall in the training activity [34,35]. Moreover, as institutions increasingly adopt electronic health records, they will need to upgrade their hardware infrastructure, which will also support the deployment of web-based training without incurring significant overhead cost. Thus, we believe that well-designed web-based training can yield an excellent ROI for healthcare systems challenged with perennial workforce training and the need for increased documentation for regulatory compliance.

Several potential limitations of this study need to be addressed. First, our study was designed to measure satisfaction and did not test for actual change in knowledge or behavior [12,36,37]. Change in knowledge and learning outcomes has been documented in many previous studies and some of these have also shown positive correlation between satisfaction and learning [6,8,11,37,38]. Second, previous studies on web-based education have found instructor-student and student-student communication to be important predictors of satisfaction [18,31]. We did not evaluate these relationships because such communication was not a part of our brief web-based training. Third, we did not directly compare web-based training with non-computer instruction (media-comparative research) because it was logistically impossible to have a valid comparison group considering the limited time frame we had at our disposal. But even well-controlled media-comparative research is difficult to generalize because observed effects cannot confidently be ascribed to any one variable [16]. Thus, many authors have suggested replacing media-comparative studies with research focusing on when to use computer-based learning (CBL), and comparing one form of CBL to another [39,40]. Finally, whether the 5% of users in our study who were dissatisfied with web-based training would have been more satisfied with traditional or blended training remains an unanswered question. Future studies can explore this question and try and determine if it is feasible to identify a group of healthcare professionals who would be better served with traditional or blended training [4].

## Conclusion

At present, the literature on web-based training in healthcare setting is limited. Our study systematically evaluated the use of web-based training across a broad spectrum of employees in a large integrated delivery network. The results suggest that web-based training can serve as a primary method of training a diverse healthcare workforce. Demographic factors and Computer Proficiency did not have a significant effect on satisfaction with web-based training. Future research should focus on measuring long-term outcomes of effectiveness, conducting formal ROI for institutions at various degrees of technology adoption and determining if it is necessary and feasible to provide alternative traditional or blended training for a targeted subgroup of health care professionals.

## Abbreviations

HIPAA: Health Information Portability and Accountability Act; ROI: return on investment; CME: Continuing Medical Education; IDN: integrated delivery network; PHI: protected health information; HR: Human Resources; CART: Classification and Regression Trees; CBL: computer-based learning.

## Competing interests

The authors declare that they have no competing interests.

## Authors' contributions

AA contributed to the conception, design, and drafting of this article. NBM led the development of web-based course, and with AKJ coordinated the survey methods and data collection. MA and HI performed psychometric validation and data analysis respectively. CMH and AJF made substantial contribution in drafting and revising the manuscript. All authors have read and approved the final version of this manuscript.

## Pre-publication history

The pre-publication history for this paper can be accessed here:


